# BioFlow: a web based workflow management software for design and execution of genomics pipelines

**DOI:** 10.1186/1751-0473-9-20

**Published:** 2014-09-18

**Authors:** Harold Garner, Ashwin Puthige

**Affiliations:** 1 Virginia Bioinformatics Institute, Washington Street 0477, Blacksburg 24061, VA, USA

## Abstract

**Background:**

Bioinformatics data analysis is usually done sequentially by chaining together multiple tools. These are created by writing scripts and tracking the inputs and outputs of all stages. Writing such scripts require programming skills. Executing multiple pipelines in parallel and keeping track of all the generated files is difficult and error prone. Checking results and task completion requires users to remotely login to their servers and run commands to identify process status. Users would benefit from a web-based tool that allows creation and execution of pipelines remotely. The tool should also keep track of all the files generated and maintain a history of user activities.

**Results:**

A software tool for building and executing workflows is described here. The individual tools in the workflows can be any command line executable or script. The software has an intuitive mechanism for adding new tools to be used in workflows. It contains a workflow designer where workflows can be creating by visually connecting various components. Workflows are executed by job runners. The outputs and the job history are saved. The tool is web based software tool and all actions can be performed remotely.

**Conclusions:**

Users without scripting knowledge can utilize the tool to build pipelines for executing tasks. Pipelines can be modeled as workflows that are reusable. BioFlow enables users to easily add new tools to the database. The workflows can be created and executed remotely. A number of parallel jobs can be easily controlled. Distributed execution is possible by running multiple instances of the application. Any number of tasks can be executed and the output will be stored making it is easy to correlate the outputs to the jobs executed.

## Background

High throughput Next Generation Sequencing techniques are producing data at a very rapid pace. The large data scale has resulted in the creation of several tools for faster processing and analysis. Bioinformatics datasets are often processed in stages. Pipelines are created so that at each stage a software package (usually a command line tool) is executed and the output produced is passed as input to the next stage.

There are multiple tools available for use at any stage in the pipeline and these tools support their own command formats. Such sequence analysis pipelines require researchers to write scripts to control the pipeline execution. Writing these scripts require knowledge of a computer programming language such as Perl, Python or bash scripting. When multiple such pipelines have to be executed, users resort to writing more scripts to control the execution order of other scripts. Users should also be able to differentiate the output files generated by various tools and isolate any failed tasks so that they can be re-executed.

Bioinformatics pipelines can be modeled as workflows where each work item is a stage (executable) in the pipeline. Workflow management software allows for the creation and execution of workflows. They are available as both command line controlled software tools that enable users to program and build custom workflows or they can contain a user-interface for predefined use cases. Web based workflow managers provide great flexibility and enable users to access them from any remote location through a browser. These allow researchers to monitor all executing tasks or create new tasks with minimal programming requirements.

Taverna [1] and Galaxy [2] are commonly used for workflow automation in bioinformatics. Both support web based workflow execution through the browser. Creating workflows in Taverna is not supported through the web interface but can be performed by installing a standalone workflow designer. Galaxy allows the addition of new tools for executing locally installed command line executables and scripts by writing a tool configuration XML file. Writing this file requires knowledge of XML [3]. bPipe [4] and Ruffus [5] are other software packages for working with workflows. bPipe is a java tool and Ruffus is available as a python module. bPipe allows users to define various execution blocks that can be joined together to create data pipelines. Ruffus module uses decorators [6] to tag functions and create an ordering of tasks. But, using them requires programming and scripting skills.

To overcome these shortcomings we have created a software program called BioFlow to greatly expand the ability for non-programmers to build sophisticated data analysis systems. BioFlow is a web-based tool for creating and managing pipelines. It has been created to be a simple and easy to use workflow management tool. The aim is to reduce the effort required in writing scripts for creating pipelines and to enable remote execution of tasks from any browser connected to the network. It has been designed so that the tools can be added quickly and hence the interface has been kept simple. Once tools are added, they can be reused and users do not need to remember command line requirements. Creating workflows is made easy by using a workflow designer. The output and status of each command is saved and this greatly simplifies the task of identifying errors and rerunning pipelines.

### Implementation

#### Architecture

Ruby on Rails is a web application development framework that favors convention over configuration [7]. This allows users to quickly understand the source code and contribute to development. It also supports a rich database of user-contributed libraries called rubygems that ease many complicated tasks. Hence, we decided to use Ruby on Rails for the server side architecture. The workflow designer on the client side is javascript intensive. jQuery is the most widely used javascript framework [8] and BioFlow uses it for handling all client side user interactions. The connections between various tools are drawn using jsPlumb [9], a library that provides a mechanism to drag and connect user interface components.

To store the data in the backend, we use a MySQL database. A developer can easily modify the configuration files to use any database of their choice. BioFlow can be deployed on any operating system supported by the Ruby on Rails runtime library, including Windows, Linux and Mac. It is deployed on an Apache server using Phusion Passenger [10].

### Model view controller

BioFlow follows a model view controller pattern for development. The three layers are kept separate and each layer can be independently modified without significantly affecting other layers.

The model layer mimics the database tables and it consists of different models for representing the tools and workflows. Each workflow consists of multiple job models and each job contains a result model. The view layer is built using HTML, CSS and jQuery. There are different views for adding tools, creating workflows and displaying outputs. Each view communicates with a different controller. This makes BioFlow modular and enables the layers to function independently of each other.

The data exchanged between the client and controller is in JSON format. Browsers are optimized for processing JSON and therefore it was a natural choice for the data format. When users create workflows in the browser, a duplicate workflow is created on the server side which is serialized and saved in the database. The order of passing outputs from one tool to another down the pipeline is stored along with the workflows.

### Background execution

By nature, bioinformatics tasks are long running. So, there are separate views for creating workflows and viewing outputs. Web applications are not tolerant of delays and so whenever requests for executing workflows are received, BioFlow only stores them in the job execution queue and returns. This job is later picked up by the background execution engine and executed. BioFlow uses a gem called delayed_job [11], which can execute tasks using job runners. A job runner is used to execute a single workflow. This allows users to control the number of parallel tasks that run on the server. This enables easy control over the load distribution to match the server’s computing capabilities. Controlling the number of parallel tasks helps in managing a server’s capacity and ensuring that it is not underutilized or overly strained.

### Distributed execution

BioFlow supports execution on multiple machines simultaneously by sharing the database among various instances. By configuring all instances to point to the same database, the workflow queue can be shared. If job runners are started on different machines, they will be able to pick up tasks from the database. But, the instances need to share the files on which the workflows are executed. This is easily achieved by using shared drives or network mounted drives. BioFlow is built for small environments and hence it allows users to manually select the server on which the task should be executed. This is useful when one of the servers is running computation intensive tasks while another server may be idle.

## Results and discussion

### Features

#### Adding command line tools to the database

**Figure 1:**
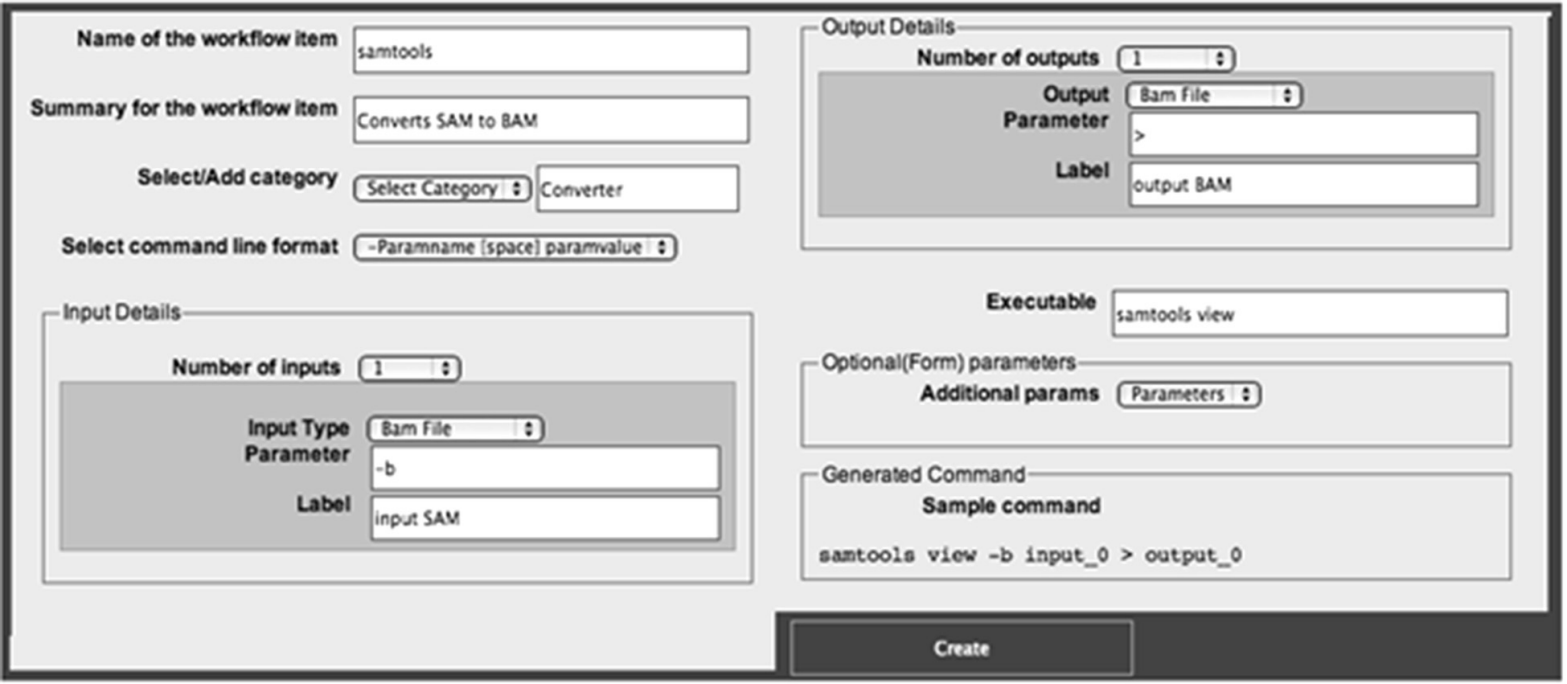
**Adding command to BioFlow.** Command Line tools are added through the Add Tools page in BioFlow. Here, it shows the addition of a tool to the category "Converter" with the command line "samtools view". It accepts one input along with the "-b" parameter and writes output to stdout which is redirected using ">" to a file. The generated sample command is also shown.

One of the main drawbacks for using graphical workflow automation tools is the difficulty in adding new tools to the software package. Researchers regularly experiment with various tools. BioFlow provides an easy and intuitive interface for adding command line tools and scripts to its database. Any user who wishes to use a new tool in a workflow has to first add it to the bioflow database.Figure 1 shows the interface that allows users to add tools to BioFlow. Complex command lines, when converted to a workflow tool, eliminate the need to remember the command line. For each tool, a name, summary and the parameters have to be provided. Optional and workflow specific parameters can be passed along during workflow execution. When adding a tool, its name, a short summary and the command line used for executing it should be specified. The tool should be installed on the server and available in a directory in user’s PATH settings. For example, the tool in Figure 1, accepts one input with the parameter "-b" and the executable name "samtools view". Parameters to generate an output file can also be specified or the output can be redirected from the standard output to a file using the redirection operator. So, in the later case, the generated command will be "samtools view –b INPUT_FILE > OUTPUT_FILE". The name of the input file is automatically passed along by the workflow executor. The name of the output file can be either specified by the user or automatically generated.

### Workflow designer

**Figure 2:**
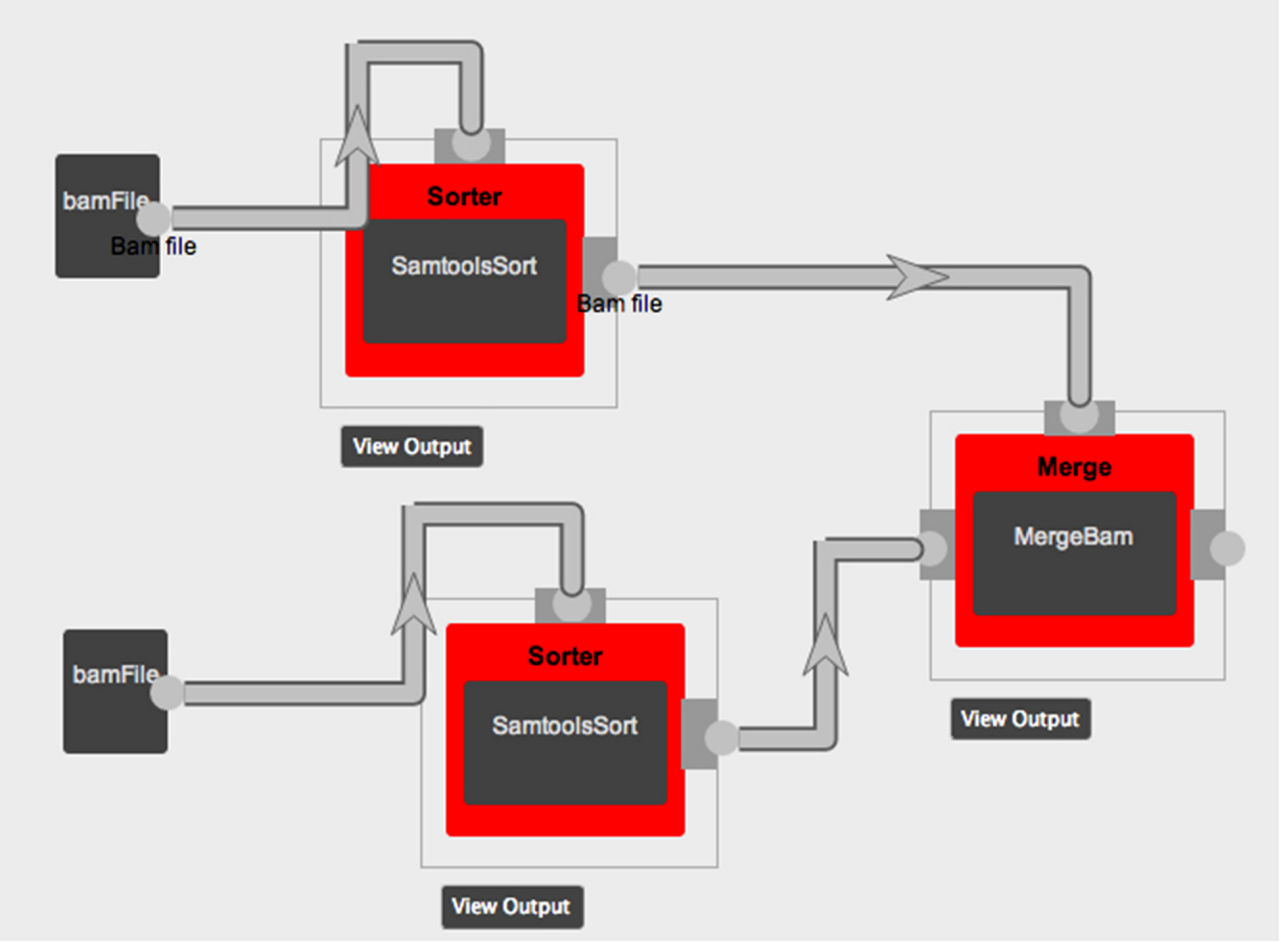
**Sample workflow creation.** This figure shows the creation of a workflow using visual programming techniques. Here, 2 input files-bam files being sorted individually using samtools and then merged to create a single bam file.

BioFlow contains a workflow designer, which allows various tools to be chained together to create workflows. The designer is divided into 3 panes – the tools pane on the left, the designer pane in the center and the optional parameters pane on the right.The tools pane lists all available tools within collapsible panels grouped together based on category. Creating workflows require users to choose the tools that are part of the workflow and interconnect them to define the flow of data in the workflow. Users can drag and drop tools to the center panel to make them part of the workflow. Each tool has input and output connections available. To create a pipeline, the output connection of one tool is connected to the input of another tool, which is the next stage in the pipeline. This creates an internal rule to pass the output file from one tool as input to the other. Figure 2 shows a sample workflow where the pipeline has 2 input files and 3 tools.

The workflows can be saved along with the parameters provided to enable reusing workflows for performing the same analysis on different input datasets.

### Viewing outputs

BioFlow has a notifications panel which automatically displays the current status of the workflow. It provides constant feedback to the user regarding the status of the workflow. Updates are displayed when the parameters are changed or the job name is modified. Since a workflow consists of multiple tools executed in succession, a notification is displayed whenever a tool starts or completes. This gives an indication to the user about the completion progress status of the workflow.

**Figure 3:**
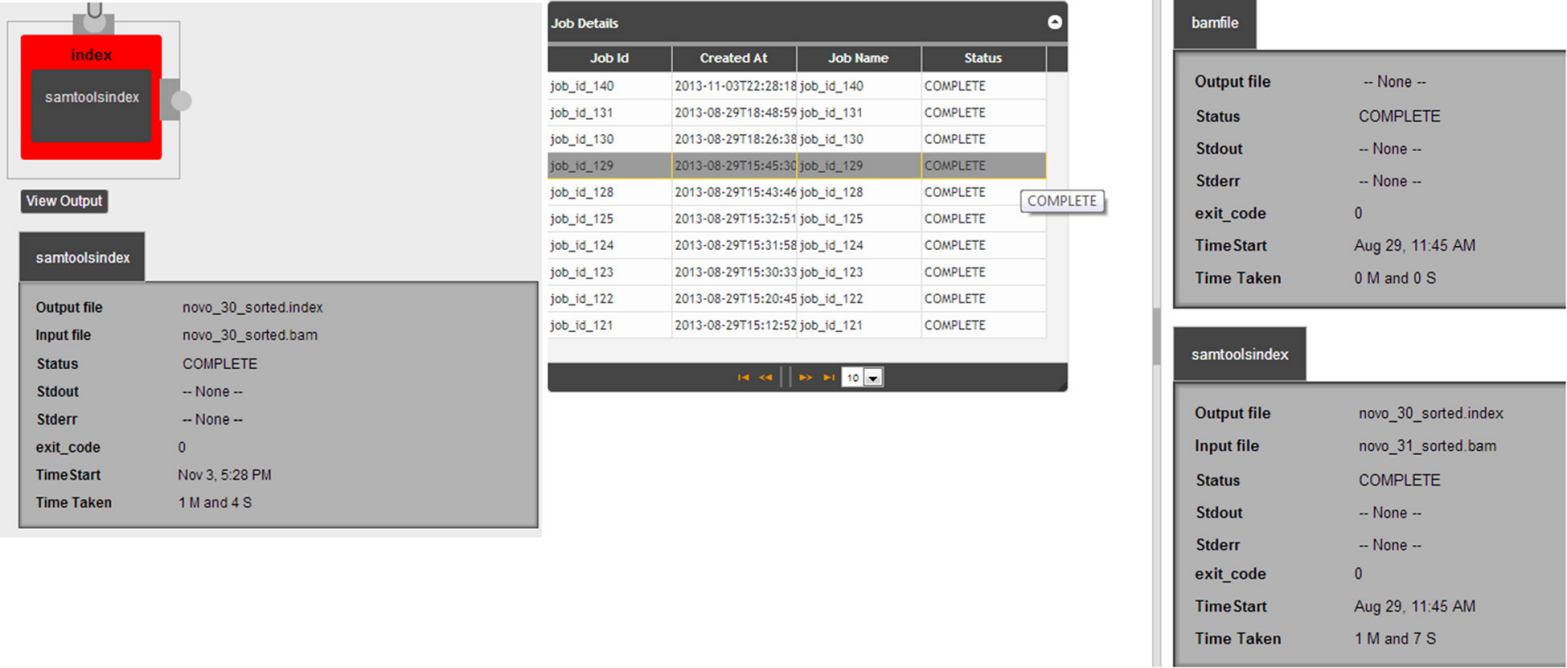
**Output below the workflow item and History of all workflows.** Bioflow provides multiple ways for viewing outputs and two of them are shown here. The image on the left shows output within the workflow designer. The output for each tool can viewed right below the tool. The image on the right shows the History of all workflows that were executed. The output of each tool is also availble in the history.

The View Output button in the workflow designer page, when clicked, shows the detailed output for the whole job. A panel slides in from the right and displays output for each tool in the workflow. The input files, output files, total time taken by the tool, exit code, stdout(standard output) and stderr(standard error) are collected and displayed in the outputs page. This allows for quick identification of any tools that have failed and helps speed debugging. All this can be viewed from within the workflow designer page.To view output of other concurrently running or completed jobs, users can browse to the view outputs page. This page displays a grid in which all jobs are listed along with their current status. Users can quickly see any failed jobs or jobs with errors and identify the root cause. Clicking on any row shows the detailed output of each individual tool. In this way, BioFlow maintains a history of all workflows that have been executed. This helps users keep track of various files generated by each pipeline. Once the task is complete, users can browse to the outputs page, note the filename and do further analysis on the file. Intermediate files are also saved. In future releases, it will be possible to modify the workflows to create unique directories for each workflow. This will further decrease the effort required for finding all required outputs created by a workflow. The outputs view as depicted in Figure 3 shows output below the tool and the history page that contains outputs of all previously executed workflows.

### Installation

Installing BioFlow requires the Ruby on Rails runtime library, MySQL database and an Internet connection to download the rubygems. After downloading BioFlow, users need to run "bundle install". This will automatically download all the required gems. Next, the config/database.yml file needs to be edited to specify the database host name. Once the database is seeded, the application can be started using "rails server". By default it runs on port 3000 and can be seen by browsing to it.

## Conclusions

BioFlow has been designed to simplify the entire process of creating and executing workflows. The simple and easy user interface for adding tools enables users to quickly add executables and scripts to BioFlow. The mechanism of visually connecting tools to build workflows allows users without programming skills to create pipelines. The functionalities provided by scripting can be done using the workflow designer. The stored outputs and history help users in debugging errors and re run only the failed pipelines. The number of parallel jobs can be controlled using job runners. This eliminates the need to write parent scripts that control other child scripts. BioFlow also provides basic distributed execution capabilities allowing users to utilize multiple servers in their environment. Hence, it greatly simplifies the task of creating, executing and tracking the outputs of bioinformatics data processing pipelines.

## Availability and requirements

The application is available at http://bioflow.vbi.vt.edu. The source code can be downloaded at the same page or at https://github.com/Bioflow/bioflow and is made available under the standard MIT license.

## Competing interests

The author(s) declare that they have no competing interests.

## Authors’ contributions

The application was designed, developed and tested by AP under the guidance of HG and other members of the lab. AP drafted the initial version of the manuscript, which was later edited after receiving comments from HG. Both authors have read and approved the final manuscript.

## Authors’ information

HG is Professor at Virginia Bioinformatics Institute, Virginia Tech and Virginia Tech Carilion School of Medicine; AP is a Masters student in the department of Computer Science, Virginia Tech.

## References

[B1] Oinn T Addis M Ferris J Marvin D Senger M Greenwood M Carver T Glover K Pocock MR Wipat A Li P Taverna: a tool for the composition and enactment of bioinformatics workflows Bioinformatics 2004 20 17 3045 3054 10.1093/bioinformatics/bth361 15201187

[B2] Goecks J Nekrutenko A Taylor J Team TG Galaxy: a comprehensive approach for supporting accessible, reproducible, and transparent computational research in the life sciences Genome Biol 2010 11 8 R86 10.1186/gb-2010-11-8-r86 20738864 PMC2945788

[B3] Galaxy Wiki – Adding tools to Galaxy http://Wiki.galaxyproject.org/Admin/Tools

[B4] Sadedin SP Pope B Oshlack A Bpipe: a tool for running and managing bioinformatics pipelines Bioinformatics 2012 28 11 1525 1526 10.1093/bioinformatics/bts167 22500002

[B5] Goodstadt L Ruffus: a lightweight Python library for computational pipelines Bioinformatics 2010 26 21 2778 2779 10.1093/bioinformatics/btq524 20847218

[B6] Ruffus http://www.ruffus.org.uk/tutorials/simple_tutorial/step1_follows.html

[B7] RubyOnRails http://rubyonrails.org/

[B8] Usage of javascript libraries http://w3techs.com/technologies/overview/javascript_library/all

[B9] jsPlumb https://jsplumbtoolkit.com

[B10] Phusion Passenger – App server for ruby https://www.phusionpassenger.com/

[B11] delayed_job https://github.com/collectiveidea/delayed_job

